# Diffusive Motion of Linear Microgel Assemblies in Solution

**DOI:** 10.3390/polym8120413

**Published:** 2016-11-29

**Authors:** Marco-Philipp Schürings, Oleksii Nevskyi, Kamill Eliasch, Ann-Katrin Michel, Bing Liu, Andrij Pich, Alexander Böker, Gero von Plessen, Dominik Wöll

**Affiliations:** 1 DWI—Leibniz Institut für Interaktive Materialien e.V., Forckenbeckstr. 50, 52074 Aachen, Germany; Marco-Philipp.Schuerings@albis.com (M.-P.S.); liubing@iccas.ac.cn (B.L.); pich@dwi.rwth-aachen.de (A.P.); 2 Lehrstuhl für Makromolekulare Materialien und Oberflächen, Forckenbeckstr. 50, 52074 Aachen, Germany; 3 Institute of Physical Chemistry, RWTH Aachen University, Landoltweg 2, 52074 Aachen, Germany; nevskyi@pc.rwth-aachen.de; 4 Institute of Physics (1A), RWTH Aachen University, 52056 Aachen, Germany; kamilleliasch@gmx.de (K.E.); michel@physik.rwth-aachen.de (A.-K.M.); 5 Functional and Interactive Polymers, RWTH Aachen University, Forckenbeckstr. 50, 52074 Aachen, Germany; 6 Fraunhofer-Institut für Angewandte Polymerforschung (IAP), Lehrstuhl für Polymermaterialien und Polymertechnologie, Universität Potsdam, Geiselbergstraße 69, 14476 Potsdam-Golm, Germany

**Keywords:** microgels, linear assemblies, in situ fluorescence microscopy, shape analysis, rotational diffusion, translational diffusion, bending stiffness, actuation

## Abstract

Due to the ability of microgels to rapidly contract and expand in response to external stimuli, assemblies of interconnected microgels are promising for actuation applications, e.g., as contracting fibers for artificial muscles. Among the properties determining the suitability of microgel assemblies for actuation are mechanical parameters such as bending stiffness and mobility. Here, we study the properties of linear, one-dimensional chains of poly(*N*-vinylcaprolactam) microgels dispersed in water. They were fabricated by utilizing wrinkled surfaces as templates and UV-cross-linking the microgels. We image the shapes of the chains on surfaces and in solution using atomic force microscopy (AFM) and fluorescence microscopy, respectively. In solution, the chains are observed to execute translational and rotational diffusive motions. Evaluation of the motions yields translational and rotational diffusion coefficients and, from the translational diffusion coefficient, the chain mobility. The microgel chains show no perceptible bending, which yields a lower limit on their bending stiffness.

## 1. Introduction

The investigation of polymer materials with spatial dimensions from a few nanometers to several microns has become an important and established research field in nano-science [1]. Improving the fundamental understanding of the mechanical, optical, electrical and magnetic properties of polymer-based nanoscale structures offers a high potential for developing future functional materials. In particular, microgels are highly suitable for creating new nanoscale superstructures, which, in the future, may lead to promising applications in diverse fields such as plastic electronics [2,3], photonics [4,5,6], biomedical applications, tissue engineering [7,8] and sensor technology [9,10,11]. In comparison to inorganic alternatives, microgels exhibit virtually unrivalled biocompatibility and are therefore gaining significant interest for drug delivery [12]. Another interesting feature is their water swelling and deswelling properties. In the swollen state, the solvent can make up more than 90% of the microgel weight. The extent of their sponge-like swelling and deswelling is determined by the type of polymer and the cross-link density. The swelling and deswelling of microgels can be triggered by external stimuli such as pH or temperature changes, and are much faster (microsecond and sub-microsecond time ranges) than swelling and deswelling of conventional macroscopic hydrogels. The reason for this difference is that the time required for diffusive transport of solvent into and out of the polymeric network scales with the network size [13].

Due to their ability to rapidly swell and deswell in response to external stimuli, microgels are potentially useful for actuation applications [14]. Individual spherical microgels are capable of substantial size changes and of generating forces during expansion and contraction. One-dimensional assemblies of interconnected microgels, which have recently been demonstrated [15], could be particularly well suited for actuation purposes. They should be capable of large contraction amplitudes along their long axis as a result of the cumulative size changes of their constituent microgels, while retaining a relatively rapid response due to the small volumes of the individual microgels. While the shape of one-dimensional microgel assemblies [15] bears similarities to those of microfilaments made of magnetic colloids and linkers [16,17,18], which have recently been studied intensively for actuation applications in microfluidics, they differ from the latter in the types of movements they should be able to generate (contractions and expansions) and in that their movements should be triggerable by temperature or pH changes.

One prerequisite for using one-dimensional microgel assemblies for actuation applications are methods to fabricate them with reproducible geometric shapes and stable links between the constituent microgels. Strategies for the synthesis of microgel assemblies can be adopted from anisotropic linear arrangements of colloids used for surface patterning and coating [19,20,21]. One such strategy is template-assisted or guided self-assembly [22,23], which gives good control over the positional order of particles in, and the dimension of, the colloidal superstructures [24,25,26,27]. Templates for guided self-assembly can be fabricated using top-down approaches (e.g., lithography and etching) or controlled wrinkling [28,29]. For example, wrinkled substrates based on poly(dimethylsiloxane) (PDMS) have resulted in an impeccable 1D alignment of microgels [15]. Using wrinkled substrates as templates, straight lines composed of single microgels as well as zigzag structures were obtained. UV cross-linking of adjacent microgels yielded one-dimensional chains of interconnected microgels, which can be released from the template by redispersing them in water [15]. In this work, the one-dimensional chains were found to have lengths of up to 20 μm and represent a unique and novel material class, which, up to this point, had never been fabricated or studied in detail.

The suitability of such microgel chains for possible actuation applications will depend, to a large extent, on their mechanical properties. Among these are the size of the force that the chain can exert during contraction along the chain axis and the velocity of the contraction. Furthermore, the chain has “passive” mechanical properties that describe the response of the structure to other forces: its tensile elasticity characterizes the response to a tensile force along the chain axis, its bending stiffness specifies the resistance to shearing forces, and its mobility gives its velocity under the combined effects of a moving force and hydrodynamic friction in the solvent. Bending stiffness and mobility are key properties that have been intensively studied in other one-dimensional systems of interest for actuation, e.g., microtubules [30], carbon nanotubes [31,32] and magnetic colloid microfilaments [16,33]. In the case of microgel chains, the values of these mechanical properties have not been determined yet. They are difficult to predict theoretically due to the granular structure of the chains and the unknown strength of the links between adjacent microgels. It is therefore necessary to determine them experimentally, to gain information on the mechanical suitability of microgel chains for actuation purposes.

In the present contribution, we study the properties of linear, one-dimensional chains of interconnected poly(*N*-vinylcaprolactam) microgels in water. They were fabricated by utilizing wrinkled polydimethylsiloaxne (PDMS) surfaces as templates and UV cross-linking the microgels. We image the shapes of the chains on surfaces and in solution using atomic force microscopy (AFM) and fluorescence microscopy, respectively. In solution, the chains are observed to execute translational and rotational diffusive motions. Evaluation of the motions yields translational and rotational diffusion coefficients and, from the translational diffusion coefficient, the chain mobility. The microgel chains show no perceptible bending, which yields a lower limit on their bending stiffness.

## 2. Materials and Methods

### 2.1. Materials

*N*-vinylcaprolactam (VCL) and acetoacetoxyethyl methacrylate (AAEM) were obtained from Sigma Aldrich (St. Louis, MO, USA), vacuum distilled under nitrogen and purified by conventional methods. The fluorescent marker rhodamine B, the initiator 2,2-azobis-2-methylpropionamidine dihydrochloride (AMPA) and the cross-linker *N*,*N*-methylene bisacrylamide (MBA) were purchased from Sigma Aldrich and used as received. Deionized water was employed as synthesis medium and solvent. Toluene for cleaning cover slides and ethanol were bought from Sigma Aldrich (anhydrous, 99.8%). A Sylgard 184 elastomer kit, made up of Sylgard 184 monomer and Sylgard 184 base, was obtained from Dow Corning (Midland, MI, USA).

### 2.2. Microgel Preparation

Poly(*N*-vinylcaprolactam) (PVCL) based microgels were synthesized as described in previously published protocols [34]. In particular, 1.877 g *N*-vinylcaprolactam (VCL, 13.5 mmol), 0.338 g acetoacetoxyethyl methacrylate (AAEM, 1.58 mmol), and 0.06 g *N*,*N*-methylene bisacrylamide (MBA, 0.39 mmol, 3 mol % based on the amount of VCL) were added to 145 mL deionized water. A three-necked flask equipped with reflux condenser and stirrer was purged with nitrogen for several minutes. After placing the monomer solution in the flask, it was stirred for 1 h at 70 °C under constant nitrogen flow. Subsequently, 5 mL of aqueous initiator solution (5 g·L^−1^) were added under constant stirring and the reaction took place for 7 h. Microgels were formed via precipitation synthesis [34]. The dispersion was then purified by a Millipore Dialysis System (cellulose membrane, molecular weight cut-off (MWCO) 100,000, Darmstadt, Germany). Monodisperse thermo-responsive microgels were obtained which exhibit a volume-phase transition (VPT) at roughly 28 °C. Below the VPTT (volume phase transition temperature) a hydrodynamic radius *r*_h_ of approximately 250 nm was determined by dynamic light scattering (DLS) at a gravimetrically determined concentration of 1.5 wt % (see Figure S1).

### 2.3. Substrate Preparation

The microgels were assembled on wrinkled substrates based on elastomeric polydimethylsiloxane (PDMS) [15]. For the wrinkle preparation, the commercially available elastomer kit Sylgard^®^ 184 was used, which contained the siloxane oligomers (Sylgard^®^ 184 Base) and a siloxane-based curing agent (Sylgard^®^ 184 curing agent), in a ratio of 10:1. The mixture was poured into a clean, flat petri dish and filled until a 3 mm thick film was obtained. The petri dish was then placed into an oven and was cured for 2 h at 80 °C after degassing overnight. With a scalpel, a 0.6 × 3 cm^2^ area was cut out from the elastomeric film, which subsequently was placed into a custom-made stretching apparatus. Preferentially, uniaxial stretching up to 130% of the initial length was applied before the substrate was placed into a plasma cleaner (Plasma active ecto 10usb-MFC), where the surface of the substrate was oxidized by air plasma under 0.2 mbar pressure. As previously shown by Genzer et al. [35], the plasma treatment time is strongly correlated to the thickness of the porous oxide layer, which in turn defines the wavelength and amplitude (i.e., the height of the structure) of the resulting sinusoidal wrinkles after relaxation. To prevent defects of the nanostructure due to substrate handling, the wrinkled elastomers were placed on adapted glass slides.

### 2.4. Fluorescence-Labelling of Microgel Assemblies

One microgram Rhodamine B was added to 50 mL PCVL/AAEM microgel dispersion in a 100 mL flask. The fluorescent marker was adsorbed into the microgel network during 24 h of stirring.

### 2.5. Formation of Microgel Assemblies and Microscopy Sample Preparation

The assembly of microgels within the grooves of the wrinkles was carried out via spin-coating. Right before adding 50 mL of the fluorescently marked microgel dispersion onto a wrinkled substrate, the wrinkles had to be activated by air plasma for 15 s at 0.2 mbar pressure to ensure a hydrophilic surface. Subsequently the sample was spin-coated at 3000 rpm for 60 s (800 rpm acceleration). Depending on the wavelength of the wrinkles, different microgel assembly formations were obtained: a 1D pearl necklace formation for 790 nm wrinkles, a zigzag formation for 910 nm wrinkles and a 2D ribbon formation for wrinkles larger than 1150 nm. To cross-link the microgel assembly, PDMS wrinkles with microgels were placed under a custom-built UV lamp (450 W Xenon short-arc, L.O.T.-ORIEL GmbH & Co. KG (Darmstadt, Germany), irradiation area *A* = 6 cm^2^; wavelength $\lambda_{\mathrm{lamp}}$ = 190‒350 nm; distance to source *δ* = 55 cm) and irradiated for 30 min. To transfer the microgel assemblies to TEM grids or glass substrates for microscopy, the sample had to be placed into the stretching apparatus again. After stretching the sample to 140% of its initial length, 10 µL water was placed onto the stretched surface. After 1 min a pipette was placed at the droplet and pressed onto the surface with a slight pressure before absorbing the droplet. The microgel assembly dispersion was transferred to TEM grids, glass slides or Si wafers for further investigations.

### 2.6. Confocal Microscopy

Microgel chains were visualized by confocal laser scanning microscopy (Leica TCS SP8, Leica Microsystems GmbH, Wetzlar, Germany, detector HyD). The samples were diluted, dropped onto a glass substrate and covered with a cover slide. Because the excitation maximum of rhodamine B is 540 nm, the microgel samples were excited with a diode-pumped solid state (DPSS) laser at 561 nm.

### 2.7. Widefield Fluorescence Microscopy

Another 561 nm DPSS laser (Cobolt Jive, 150 mW, Cobolt AB, Solna, Sweden) was used for the excitation of the dye molecules in the widefield fluorescence microscopy. The laser intensity was adjusted by neutral density filters to ca. 1–2 kW/cm^2^. The laser was coupled into a multi-mode fiber (UM22-600 Thorlabs, NA 0.22 ± 0.02, 0.6 mm core diameter), which was shaken to destroy the coherence and suppress interference effects in oder to obtain homogeneous sample illumination. The end of the fiber was imaged onto a ca. 40 × 40 µm^2^ area of the sample using a 100×/1.49 NA oil immersion objective (UAPON100×OTIRF, Olympus, Olympus Deutschland GmbH, Hamburg, Germany). The samples were placed on an *xyz*-piezo table (P-545.3R7, Physik Instrumente, Karlsruhe, Germany), which fits into a commercial microscope (Olympus IX83, Olympus Deutschland GmbH, Hamburg, Germany). The fluorescence was collected using the same objective and was spectrally separated from the excitation laser light by a quad line beamsplitter (zt405/488/561/640rpc, AHF Analysentechnik, Tübingen, Germany), further magnified by two lenses (*f*_1_ = 200 mm AC254-200-A, Thorlabs and *f*_2_ = 400 mm AC254-400-A, Thorlabs, Thorlabs, Dachau, Germany) and imaged onto the chip of an EMCCD camera (Andor iXON Ultra 897, Andor Technology Ltd., Belfast, UK). To increase the S/N-ratio, a notch filter (E grade, 561 nm, AHF Analysentechnik) and a bandpass filter (Brightline HC 617/73, AHF Analysentechnik) were placed between the two lenses, and a cleanup filter (z561/10, AHF Analysentechnik) was placed in front of the laser. The total magnification was 200-fold, resulting in an image with 77 nm per pixel of the CCD camera. All measurements were performed at 294 K with an integration time per frame of 50 ms.

### 2.8. Analysis of Microgel Assembly Conformation

The shape fluctuations contour analysis based on widefield images was performed with the Easyworm software package [36]. Parametric splines were used to approximate the contour, while the persistence length was evaluated within the framework of a worm-like chain model for a semi-flexible polymer [36,37].

### 2.9. Dynamic Light Scattering

The hydrodynamic radius and the VPTT of the microgel dispersion were measured by dynamic light scattering (ALV Goniometer with scattering detection at an angle of 90°, ALV-Laser Vertriebsgesellschaft m-b.H., Langen/Hessen, Germany).

### 2.10. Atomic Force Microscopy (AFM)

Wrinkles with aligned microgels and Si wafers with dispersed microgel assemblies were analyzed by AFM (Veeco Dimension ICON from Bruker, Billerica, MA, USA) with OTESPA tips (278–357 kHz, 12–103 N·m^−1^) in tapping mode. Nanoscope 8.10 (Build R1.60476) software was used to analyze the AFM data.

### 2.11. Transmission Electron Microscopy (TEM)

Ten microliters of the microgels assembly dispersion were placed onto a carbon-coated copper grid, dried and examined by TEM (Zeiss Libra 120, Carl Zeiss NTS GmbH, Oberkochen, Germany) at 80 kV in bright field mode.

## 3. Results and Discussion

### 3.1. Synthesis of Microgel Assemblies

**Figure 1:**
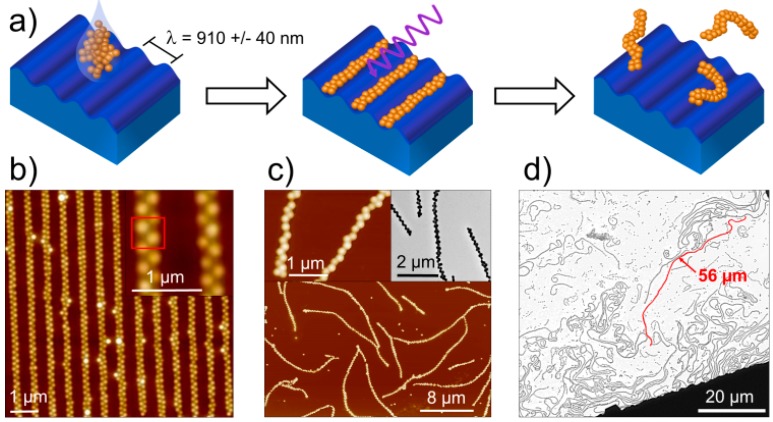
Sample preparation scheme and corresponding AFM and TEM images: (**a**) alignment of microgels (represented by orange spheres) in the wrinkle grooves (blue trenches) via spin-coating, UV irradiation (wavy arrow) of microgel assemblies and redispersion of cross-linked microgel assemblies; (**b**) AFM image of microgels aligned in a zigzag formation within the wrinkle grooves and magnification of the microgel overlap; (**c**) AFM and TEM images of redispersed microgel chains transferred onto a substrate (Si wafer and TEM grid, respectively); and (**d**) TEM image of microgel chains; maximum lengths are beyond 50 µm.

Poly(*N*-vinylcaprolactam) (PVCL) based microgels with a hydrodynamic radius *r*_h_ of approximately 250 nm and a volume phase transition temperature (VPTT) of 28 °C were synthesized as described in previously published protocols [34]. The wrinkled PDMS surfaces described above, were used for guided self-assembly of the microgels. The wrinkle wavelength λ, i.e., the distance between two adjacent grooves (Figure 1a), is controlled by the duration of the plasma treatment. To ensure an appropriate microgel alignment within the grooves, λ has to be in the same order of magnitude as the diameter of the microgels [15]. The PVCL microgels were assembled onto the wrinkled PDMS substrates by spin-coating (Figure 1a). Figure 1b shows a zigzag alignment within the wrinkles. In contrast to *N*-isopropylacrylamide (NiPAAm) microgels [15], the alignment of PVCL microgels is solely based on the dewetting process during spin-coating. This is because PVCL microgels are sterically stabilized and charges causing electrostatic interactions with the negatively charged silanol groups of the oxidized PDMS surface are absent. Furthermore, the acetoacetoxyethyl methacrylate (AAEM) involved in the cross-linking plays a major role by increasing the rigidity of the PVCL microgels through formation of a hydrophobic and rigid core, which supports an alignment through spin-coating.

After alignment, the assemblies were cross-linked by UV irradiation in a wavelength range from 190 to 310 nm to ensure inter-particle connectivity (Figure 1a). The microgels used in the present study exhibit heterogeneity in their structure due to the predominant localization of AAEM units (β-diketone groups) in the core. However, a small fraction of β-diketone groups can also be localized in the microgel coronae. The reason for this is the different polarity of the keto- and enol-forms of AAEM that considerably influences the incorporation of AAEM units into the microgel structure during precipitation polymerization. It is reasonable to assume that a considerable amount of the keto-form of AAEM, which readily forms hydrogel bonds with water molecules, will be incorporated in the microgel corona. Considering the fuzzy surface of microgels and the close alignment of the microgels in the chains before UV irradiation justifies the assumption that β-diketone groups of neighboring microgels will be localized in close proximity to each other, providing sufficient conditions for inter-particle crosslinking. It has been reported in the literature that UV irradiation induces photo-tautomerisation of β-diketones in water, thus influencing their reactivity [38]. We assume that the β-diketone groups present in the keto or the enol form attached to the polymer chains of the microgel can act as substrates for the radical formation reaction under UV irradiation in the presence of oxygen and water. The activity of β-diketones as radical mediators was already observed in enzymatic radical polymerization [39].

The cross-linked microgel assemblies were redispersed into a small amount of water and subsequently transferred onto previously cleaned silicon wafers or glass cover slips. Analysis of the samples via AFM and TEM proved that stable microgel chains with a granular structure and lengths up to 56 µm, i.e., with lengths larger than those previously reported [15], were obtained (Figure 1c,d). The fact that such large lengths can be found after transferral means that most of the links between adjacent microgels survive the forces effective during redispersal in water and redeposition. However, it has to be noted that in our current approach the chain length cannot be controlled and depends on the density of the defects (i.e., cracks that are perpendicular to the wrinkles) in the template surface. The granular structure of the chains, which can be clearly seen in the insets of Figure 1c, is determined by the surface curvatures of the constituent microgels and their center-to-center distances.

Depending on the wavelength of the wrinkles, microgel chains with pearl necklace, zigzag, and ribbon-like formations, respectively, are obtained, as shown in Figure S2. By increasing the wavelength of the templates, the width of the assemblies increases while their height remains unchanged (i.e., the height of one microgel). The reason for this phenomenon lies in the fact that the PVCL microgels are sterically stabilized, which avoids agglomeration in dispersion and hinders stacking on top of each other during spin-coating. A high degree of overlap between the soft microgels is necessary for good stability of the chains. In the pearl necklace formation, microgels are only connected via a few filaments, and there are many potential breaking points, as can be seen in the magnified TEM image in Figure S2a of the Supplementary Materials. The high mass of the ribbon-like chains leads to faster precipitation. In contrast, the zigzag alignment combines a high degree of overlap between adjacent microgels and a relatively low mass. Therefore, the investigations of free-moving microgel chains described below were all performed with microgels aligned in the zigzag formation.

### 3.2. Visualization of Microgel Assemblies with Fluorescence Microscopy

In what follows, we study the motion of the microgel chains in solution with the aim of extracting information on the mechanical properties of the chains. We focus here on two parameters which describe the response of the chains to different kinds of forces that are effective during motions in a liquid: the mobility *μ*, which gives the velocity of an immersed object under the combined effects of a moving force and hydrodynamic friction, and the bending stiffness *κ*, which describes the resistance to shearing forces. Experimentally we follow an approach similar to those of Han et al. [40] and Fakhri et al. [31], in which the Brownian motions of ellipsoidal microparticles and single-walled carbon nanotubes, respectively, were measured using fluorescence microscopy. The Brownian motions constitute the response of the system to random fluctuations in the liquid. They can be evaluated to yield the values of the mobility and bending stiffness. The values of *μ* and *κ* thus determined also quantify the response to steady forces and are therefore important for the description of actuated motion.

**Figure 2:**
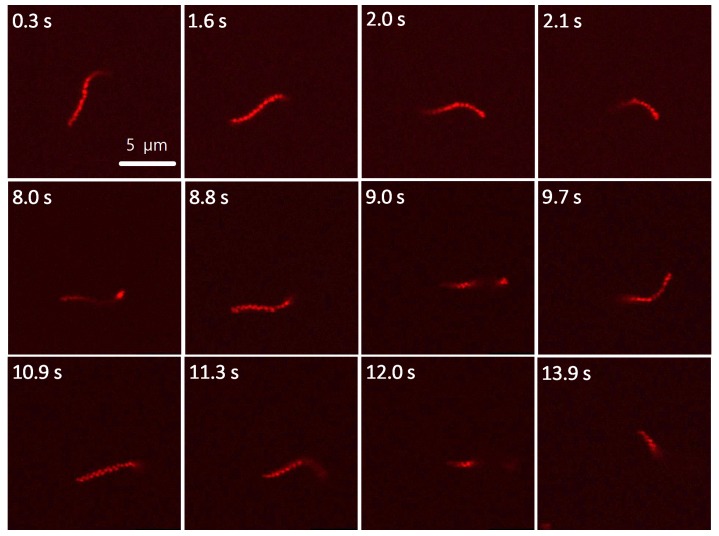
Freely moving Poly(N-vinylcaprolactam) (PVCL)/acetoacetoxyethyl methacrylate (AAEM) microgel chain (with adsorbed rhodamine B) observed by confocal fluorescence microscopy. Additional examples of confocal microscopy images of freely moving microgel chains are shown in Figure S7 of the Supplementary Materials. A representative movie is found as Movie 4 of the Supplementary Materials. Scale bar 5 µm.

**Figure 3:**
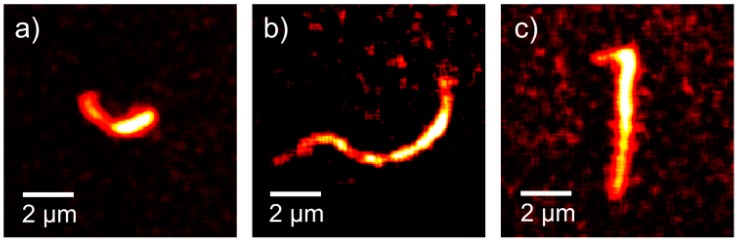
(**a**–**c**) Typical shapes of microgel chains observed in widefield fluorescence microscopy measurements. The three chains exhibit a C-shape, S-shape and L-shape, respectively. The following contour lengths L were obtained by Easyworm [36]: (**a**) *L* = 3.8 µm; (**b**) *L* = 11.4 µm; and (**c**) *L* = 7.6 µm. The images were filtered as described in the Supplementary Materials, where the corresponding movies can also be found. The corresponding Movies 1–3 can be found in the Supplementary Materials.

To visualize the microgel chains in our fluorescence microscopy experiments, rhodamine B (RhB) was added to the microgels as a fluorescent marker. The resolution of optical microscopy is restricted to the Abbe limit, which means that structures below approximately half the wavelength of the observation light cannot be resolved. Superresolved fluorescence microscopy methods allow for a circumvention of the diffraction limit [41], but are restricted by their time resolution. Thus, we used confocal microscopy and wide-field fluorescence microscopy to observe the fluorescence-labeled microgel assemblies. The former possesses a higher spatial resolution, in particular in *z*-direction. This allows for resolving individual microgels in the microgel assembly, as demonstrated in Figure 2. This improved *z*-resolution, however, has the disadvantage that the *z*-position of the objective has to be adapted continuously to the three-dimensional motion of the chains in water. This problem is particularly challenging for the observation of fast moving chains. Thus, only few movies taken using confocal microscopy were usable for further analysis. In contrast, wide-field fluorescence microscopy is less sensitive to the motion of microgel chains along the *z*-direction. Despite the higher background and the fact that individual microgels within the chain could not be resolved (Figure 3), long movies of single microgel chains moving in water were obtained with this method and could be readily analyzed. In order to observe the free motion of microgel chains within the liquid, they had to be prevented from adsorbing on surfaces. This was achieved by spin-coating a thin PVCL layer (*M*_n_ ~ 15,000 g·mol^−1^; *c* = 10^−4^ mol·L^−1^) onto the substrate surface. Subsequently, a small amount of cross-linked zigzag microgel chains dispersed in water were transferred onto the polymer-functionalized glass substrate, and confocal or wide field fluorescence microscopy images of the dispersion were taken, respectively.

A number of freely moving microgel chains were observed using this approach, providing information on the diffusion behavior of the supramolecular structures. Examples of image series and movies can be found in the Supplementary Materials. Detailed information about the motion and the flexibility of the chains was extracted via image analysis as discussed in the following section.

### 3.3. Analysis of the 2D Projections of Microgel Chains

Since the time resolution of confocal microscopy does not allow sufficiently rapid 3D scanning and widefield fluorescence microscopy does not resolve the axial *z*-direction to better than a few micrometers, we were only able to record 2D projections of the 3D orientation of the microgel chains. In what follows, we describe how the 2D projections were analyzed.

In order to gain an understanding of the shapes of the synthesized microgel chains and their dynamics, we analyzed the recorded movies using image analysis tools provided by the Easyworm software package [36]. Using these tools, the shape of each analyzed microgel chain was determined by fitting it to a spline after appropriate image filtering.

The observed microgel shapes can be categorized into three different groups: C-, S- and L-shapes (see Figure 3). The curvature of the C- and S-shapes may be caused by inhomogeneous cross-linking as a consequence of attenuation of the UV radiation by those regions of the microgels that face the irradiation source during cross-linking. Thus, the swelling properties of opposite sides of the microgel assemblies may differ. This could explain the C-shape and, assuming slight rotations around the long axis of the chain during the irradiation, also the S-shape. A possible origin of the unusual L-shaped chains may be the lateral cracks in the wrinkled substrate.

**Figure 4:**
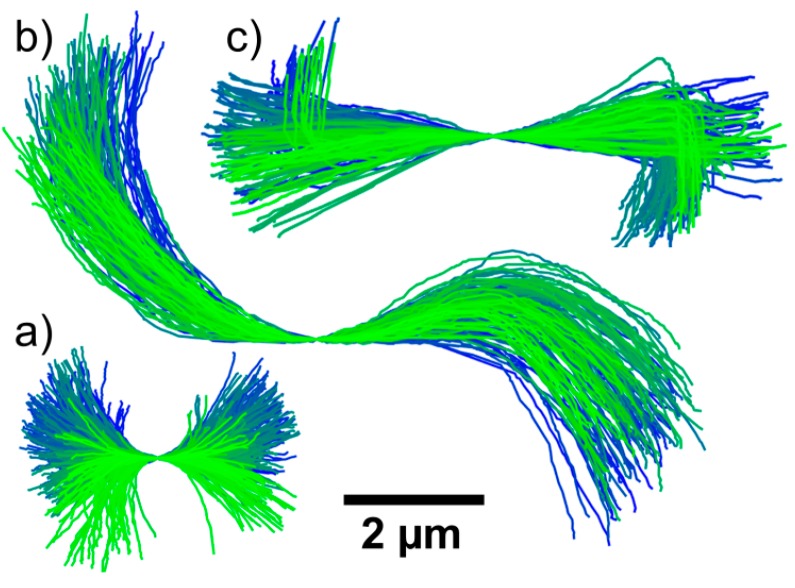
(**a**–**c**) Temporal evolution of the 2D projections of the microgel chains shown in the fluorescence microscopy images in Figure 3. For a clearer representation, the centers of the chains were positioned in the same points and the 2D projections of the chains were rotated in-plane so that the slope in this point equals zero. The color variation from blue to green represents the evolution over the frame sequence.

As shown in Figure 2, the different 2D shapes in the microscopy images were observed to vary over time. This variation is caused by Brownian motion, as will be confirmed in the following subsections. More examples of such variations are given in Figure 4, which shows the splines for the three microgel chains from Figure 3a–c. For a clearer representation, the centers of the chains have been positioned in the same point and the splines rotated in-plane so that their slope in this point equals zero; these manipulations effectively remove effects of the center-of-mass motion and in-plane rotation from the representation. A careful analysis shows that the remaining variations in length and shape of the splines, i.e., their apparent shortenings and changes of curvature, are consistent with out-of-plane rotations of the chains, i.e., rotations around axes in the object plane. In contrast, their 3D conformation does not seem to change significantly within our observation time window. This implies that they are much stiffer than the single-walled carbon nanotubes studied by Fakhri et al. [31], which showed considerable bending at comparable lengths but much smaller thicknesses.

From the 2D projections obtained by both fluorescence microscopy methods, we determined characteristic geometric parameters for each analyzed chain using the Easyworm software package [36]. In particular, the contour length *L*_xy_ of the 2D projection of the chain in each frame was determined from the spline fit. Due to the perspective-induced shortening of the chain during 3D rotation, the values of *L*_xy_ can vary from frame to frame. To determine the contour lengths *L* of the chain itself, we selected for each chain the maximum value of *L*_xy_, corresponding to the frame with the minimum perspective-induced shortening. This procedure yields, for example, the following contour lengths for the microgel chains shown in Figure 3a–c: (a) *L* = 3.8 µm; (b) *L* = 11.4 µm; and (c) *L* = 7.6 µm.

The fact that the microgel chains show no perceptible bending dynamics on the time scale of the experiment means that the persistence lengths of the chains, $L_{p},$ are much greater than their contour lengths, $L_{p}\gg L$. For the bending stiffness $\kappa =k_{B}T\cdot L_{p}$, this implies that $\kappa \gg k_{B}T\cdot L$, where $k_{B}$ is the Boltzmann constant. For example, this inequality yields 5 × 10^−26^ N·m^2^ as a rough lower limit on the bending stiffness of the chain with a contour length of *L* = 11.4 µm in Figure 3b and Figure 4b. At present no comparison of this value with theory is possible due to the unknown strength of the links between adjacent microgels. It is interesting to note that this lower limit lies in the order of magnitude range spanned by the κ values that were determined for chains of linked magnetic colloids by Biswal et al. [16] and Goubault et al. [17] (κ between 1.5 × 10^−26^ and 1.1 × 10^−21^ N·m^2^). In the magnetic-colloid chains, κ depends exclusively on the kind of molecule used to link the colloids, because the latter are not deformable.

### 3.4. Translational Diffusion of Microgel Chains

**Figure 5:**
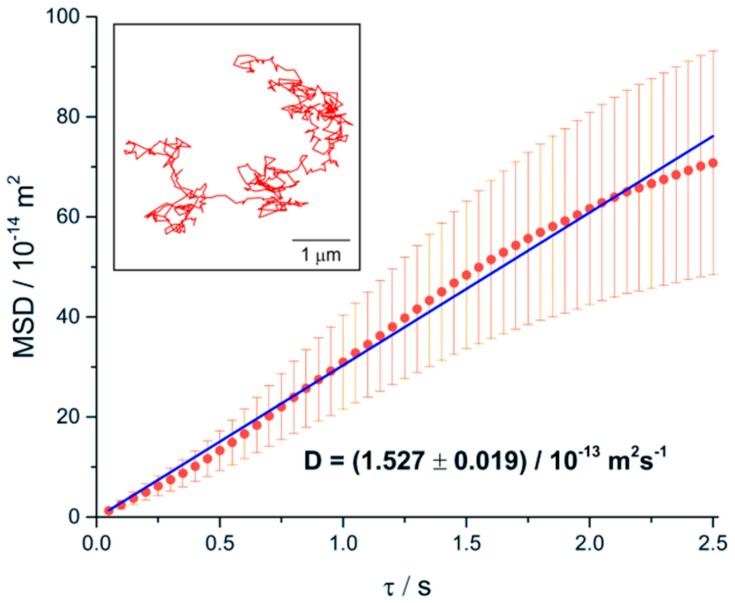
Temporal evolution of the mean square displacement of the center-of-mass of the microgel chain from Figure 3a and Figure 4a. Blue line: linear fit. Inset: single track of the center-of-mass. The corresponding diagrams for the other two examples of microgel chains in Figure 3 and Figure 4 are shown in Figures S3–S6.

The translational motion of the microgel chains was studied by analyzing in each frame the center-of-mass position of the projection of the chain. This position was obtained from the shape of the projection using auxiliary points homogeneously distributed over each spline. An example of the temporal evolution of the mean square displacement $\langle \Delta x^{2}+\Delta y^{2}\rangle$ of the center-of-mass of the projection is presented in Figure 5 for the chain shown in Figure 3a and Figure 4a (see the Supplementary Materials for an excerpt from the movie of the motion of this chain). The time dependence of the mean square displacement can be approximately described by a linear fit (blue line), showing that the microgel chain diffuses normally due to Brownian motion. Using the relation $\langle \Delta x^{2}+\Delta y^{2}\rangle =4D_{t}\cdot t$, which is valid for 2D projections of 3D diffusive motions, [42] the isotropic translational diffusion coefficient is determined to be $D_{t}$ = 0.15 μm^2^·s^−1^ from the slope of the fit in Figure 5.

It is instructive to compare this experimental result to theory using a model for the translational and rotational diffusion of cylinders developed by Broersma [43,44]. Such models have been experimentally validated for rod-like particles [32,45,46,47]. The translational and rotational diffusion coefficients of a cylindrical rod are predicted to be

(1) $$D_{i}^{\left(th\right)}=\frac{k_{B}T}{\pi \eta L}a_{i}\left(\ln\left(L/d\right)+\nu_{i}\right)$$

where the index *i* = ∥ stands for translational diffusion along the direction of the rod axis, *i* = ⊥ for translational diffusion in a sidewise direction, *i* = t for isotropic translational diffusion and *i* = R for rotational diffusion, i.e., diffusive reorientation of the rod axis. *T* is the temperature, η is the viscosity of the solution, *L* is the rod length, and *d* is the rod diameter. The $a_{i}$ are given by $a_{∥}=1/2,\;a_{⊥}=1/4,\;a_{t}=1/3$ and $a_{R}=3/L^{2}$. The end correction coefficients $\nu_{i}$ are polynomials in ${\left(\ln2\left(L/d\right)\right)}^{-1}$ and listed by Broersma [44]. The comparison between the model calculation and our experiment is complicated by the shape of our microgel chains, which is not strictly cylindrical. In particular, the zigzag arrangement of microgels in the assemblies studied here (cf. Figure 1b,c) means that the cross-section of the chains perpendicular to their axis is anisometric with dimensions $d_{1}\approx 0.50$ µm (i.e., the diameter of an individual microgel) and $d_{2}\approx 0.93$ µm. To simplify the comparison between calculation and experiment, we determine an effective rod diameter *d* by fitting Equation (1) to the measured value of the isotropic translational diffusion coefficient $D_{t}$ and using *d* as a fit parameter. For example, inserting *T* = 293 K, η = 1.00 mPa s and *L* = 3.8 µm into Equation (1), the fit yields a perfect match with the measured value $D_{t}$ = 0.15 µm^2^·s^−1^ for *d* = 1.08 µm. Fits for the other microgel chains studied here yield *d* values within 10% of 1.08 µm. This effective diameter is close to $d_{2}\approx 0.93$ µm, the thick dimension of the cross-section measured by AFM.

One of the goals of the present work is to determine a value for the translational mobility. The isotropic translational mobility $\mu_{t}$ can be calculated from $D_{t}$ using the Einstein relation $\mu_{t}=D_{t}/k_{B}T$. Inserting our experimental result $D_{t}$ = 0.15 µm^2^·s^−1^ into this equation yields $\mu_{t}$ = 3.8 × 10^7^ s·kg^−1^. It is important to note that the isotropic translational mobility $\mu_{t}$ obtained in this way describes the translational motion of the microgel chain only in conditions in which the orientation of the chain varies randomly. If, in contrast, a situation with a fixed orientation of the chain is of interest, e.g., in an actuation application, then the translational mobilities along and perpendicular to the direction of the rod axis, $\mu_{∥}$ and $\mu_{⊥}$, are relevant. While our experiment is not able to determine them separately, they can be calculated by inserting the effective diameter *d* = 1.08 µm, which has been determined above, into Equation (1) for *i* = ∥ and ⊥, and inserting the resultant values of $D_{∥}^{\left(\mathrm{th}\right)}$ and $D_{⊥}^{\left(\mathrm{th}\right)}$ into the Einstein relation $\mu_{i}=D_{i}/k_{B}T$. Thus, we obtain $\mu_{∥}$ = 4.1 × 10^7^ s·kg^−1^ and $\mu_{⊥}$ = 3.6 × 10^7^ s·kg^−1^.

### 3.5. Rotational Diffusion of Microgel Chains

The rotational diffusion of microgel chains can be determined from the temporal evolution of orientations in successive widefield fluorescence microscopy frames. As discussed above, in our current study, we were only able to determine and analyze 2D projections, rather than the full 3D orientation. Nevertheless, the rotational diffusion coefficient $D_{R}$ can be estimated from the temporal evolution of such 2D projections [48]. For this purpose, the orientational correlation function for the polar angle ψ of the 2D-projected orientations at different time delays τ is fitted according to

(2) $$\langle \cos\psi \rangle \left(\tau \right)\approx e^{-2D_{R}\tau }$$

**Figure 6:**
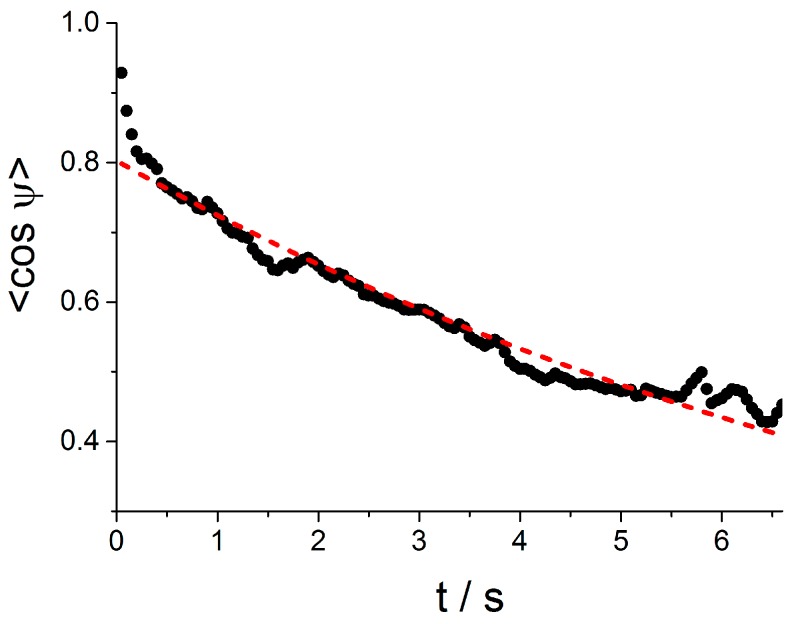
Temporal evolution of the mean square displacement of the center-of-mass of a microgel chain. Blue line: linear fit. Inset: single track of the center-of-mass.

The temporal evolution of the orientational correlation function of the microgel chain from Figure 5 is shown in Figure 6. Fitting an exponential function according to Equation (2) yields a rotational diffusion coefficient $D_{R}$ = 0.10 s^−1^.

For a comparison with theory, we use Equation (1) with *i* = R and the end correction coefficient for rotational diffusion from Broersma [44]. Due to the anisometric cross-section of the microgel chain, we follow the same approach as above and determine an effective rod diameter *d* by fitting Equation (1) to the measured value $D_{R}$ = 0.10 s^−1^, using *d* as a fit parameter. The fit yields *d* = 0.46 µm, a result close to $d_{1}$ ≈ 0.50 µm, the thin dimension of the cross-section. The discrepancy between this result and that obtained from the fit to $D_{t}$ (*d* = 1.08 µm) may be attributable to the curved shape, non-circular cross section, and granular structure of the microgel chain, which are not contained in the cylinder model that was used here. Fortunately, the discrepancy is not of critical importance, because the dependencies of $D_{t}^{\left(\mathrm{th}\right)}$ and $D_{R}^{\left(\mathrm{th}\right)}$ on the value of *d* are relatively weak in this range of diameters; when *d* is varied freely between the two fit results, i.e., in the range 0.46–1.08 µm, the model calculation gives that $D_{t}^{\left(\mathrm{th}\right)}$ varies between 0.23 and 0.15 µm^2^·s^−1^, and $D_{R}^{\left(\mathrm{th}\right)}$ between 0.10 and 0.05 s^−1^. In particular, all values in this $D_{t}^{\left(\mathrm{th}\right)}$ range still lie reasonably close to the experimental result $D_{t}$ = 0.15 µm^2^·s^−1^. Similarly, the uncertainty in the effective diameter *d* translates into relatively narrow ranges μ = 3.8 × 10^7^–5.8 × 10^7^ s·kg^−1^, $\mu_{∥}$ = 4.1 × 10^7^–6.5 × 10^7^ s·kg^−1^ and $\mu_{⊥}$ = 3.6 × 10^7^–5.4 × 10^7^ s·kg^−1^ of the calculated isotropic, lengthwise and sidewise translational mobilities, respectively.

## 4. Conclusions

In the present contribution, we have studied the properties of linear chains of poly(*N*-vinylcaprolactam) microgels dispersed in water. They were fabricated by utilizing wrinkled PDMS surfaces as templates and UV cross-linking the microgels. We have imaged the shapes of the chains on surfaces and in solution using AFM and fluorescence microscopy, respectively. In solution, the chains have been observed to execute translational and rotational diffusive motions. Evaluation of the motions has yielded translational and rotational diffusion coefficients that are in reasonably good agreement with the results of a model calculation for cylindrical rods in solution. From the translational diffusion coefficient, the chain mobility has been calculated. The microgel chains show no perceptible bending, which yields a lower limit on their bending stiffness.

Our work, in which the free motion of microgel chains in solution has been evaluated to gain information on their mobility and bending behavior, is a step towards a comprehensive characterization of the mechanical properties of these microgel assemblies. The chains studied here consist of dozens or even hundreds of discrete submicron units strung together by cross-linking. As such, they have few parallels in nanotechnology, the most notable being the microfilaments made of linked magnetic colloids that have recently been studied for actuation applications in microfluidics [16,33]. In comparison to the latter, microgel chains may have specific potential for possible actuation applications that necessitate contraction and expansion along the chain axis and the possibility of triggering movements by temperature and pH changes. Before this potential can be realized, however, more research is needed, with particular attention to controlling the bending stiffness of the chains and their overall shapes and lengths, as well as investigating their contraction and the forces exerted by them.

## Supplementary Materials

The following are available online at www.mdpi.com/2073-4360/8/12/413/s1.
