# Supplementary material for: Diffusive Motion of Linear Microgel Assemblies in Solution
Source: Polymers (Basel). 2016 Nov 29;8(12):413. doi: 10.3390/polym8120413 (PMC6432013; doi:10.3390/polym8120413)
Supplement: Supplementary file 1 [file polymers-08-00413-s001.zip › polymers-158299-supplementary.pdf]

Supplementary Materials: Diffusive Motion of Linear Microgel Assemblies in Solution

Marco-Philipp Schürings, Oleksii Nevskyi, Kamill Eliasch, Ann-Katrin Michel, Bing Liu, Andrij Pich, Alexander Böker, Gero von Plessen and Dominik Wöll

1. Temperature-Dependent DLS Measurements

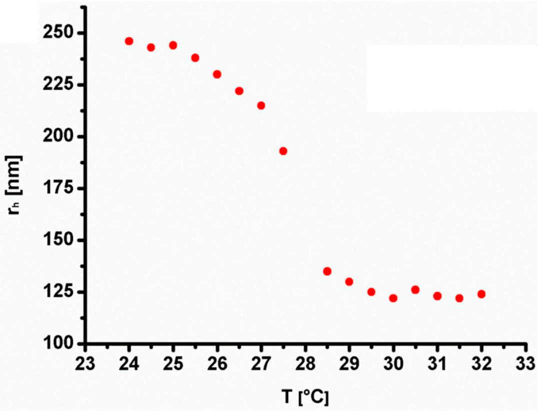

Figure S1. DLS data of VCL/AAEM microgels showing the VPT of the synthesized sample.

2. AFM Images of Microgel Chains Obtained on Substrates of Different Wavelength

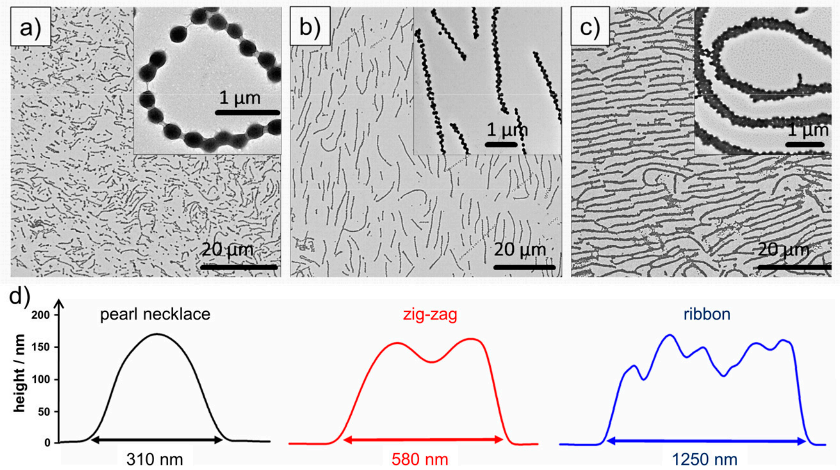

Figure S2. TEM images of microgel chains after alignment in wrinkles and transfer onto a substrate: (**a**) pearl necklace formation based on 790 nm wrinkles; (**b**) zig-zag formation (910 nm wrinkles) and (**c**) ribbon formation (wrinkles larger than 1150 nm); (**d**) lateral cross-section of one representative microgel chain of each conformation.

3. Supporting Movies

The following widefield fluorescence microscopy movies can be found in the supporting material:

Movie 1: C-shaped microgel chain (a)
Movie 2: S-shaped microgel chain (b)
Movie 3: L-shaped microgel chain (c)

All movies are played in real-time. For better visibility, they were smoothed by a mean filter to reduce noise and their background corrected by a Matlab filter performing morphological opening. The blue dot marks the center-of-mass of the microgel chains, the line through the dot represents their orientation.

For comparison, a movie obtained by confocal microscopy is shown as Movie 4. This movie has a slightly better resolution. However, the higher resolution in *z* causes parts of the microgel chain which are more than one micrometer out of focus to disappear completely.

4. Translational and Rotational Analyses

The movies presented above were analysed according to the procedure described in the paper and, for Movie 1, shown in Figures 5 and 6.

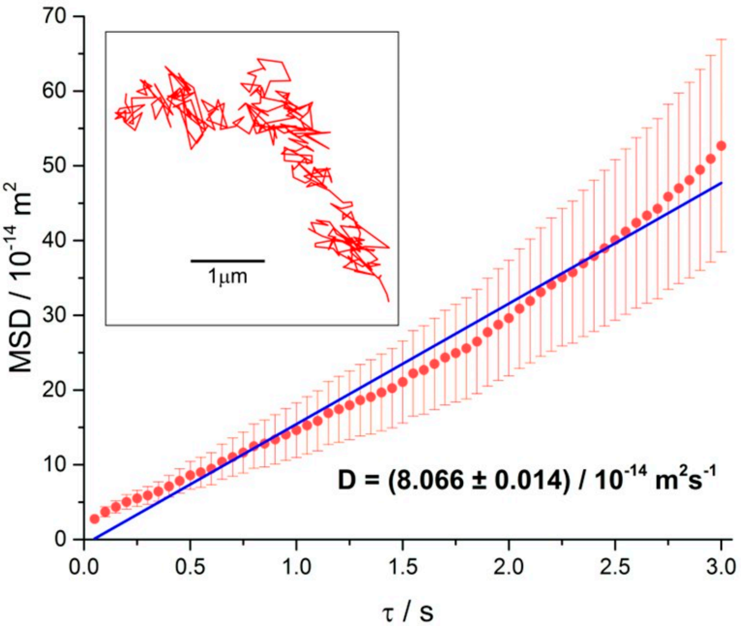

Figure S3. Temporal evolution of the mean square displacement for the center-of-mass of microgel chain b (see Movie 2). Inset: single track of the center-of-mass of the same microgel chain.

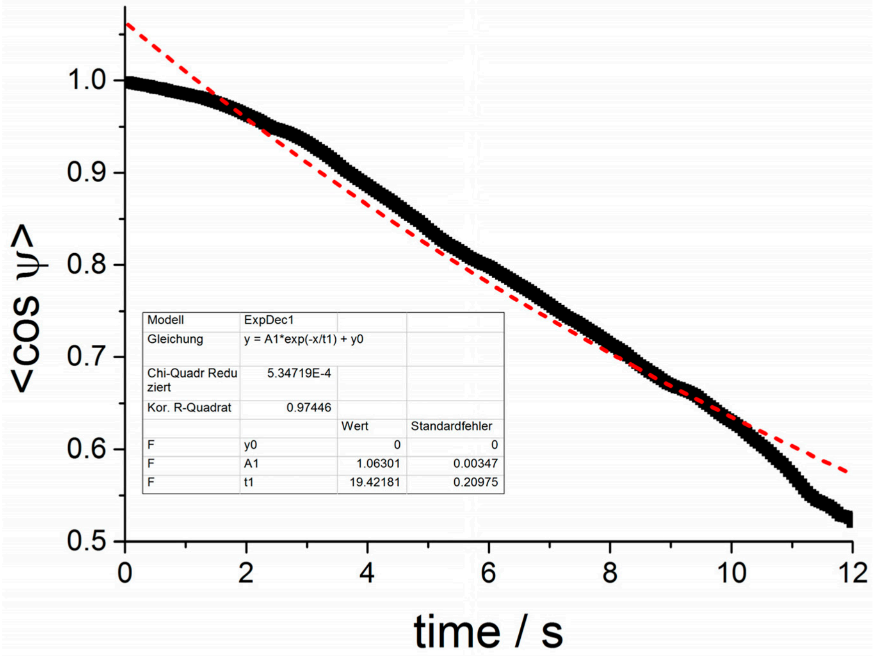

Figure S4. Temporal evolution of the average angle ψ of 2D-projected orientations for the microgel chain b (see Movie 2).

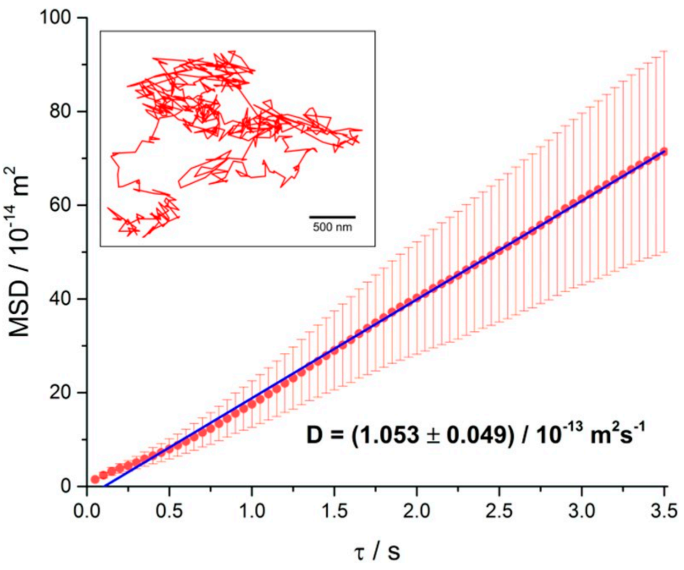

Figure S5. Temporal evolution of the mean square displacement for the center-of-mass of microgel chain c (see Movie 3). Inset: single track of the center-of-mass of the same microgel chain.

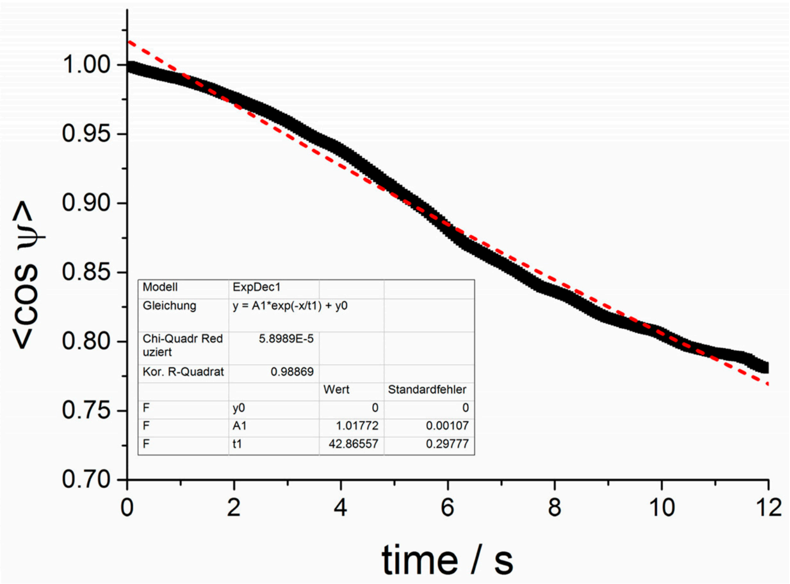

Figure S6. Temporal evolution of the average angle ψ of 2D-projected orientations for microgel chain c (see Movie 3).

A comparison between the rotational and translational diffusion of the microgel chains presented in the movies described above can be summarized as shown in the following table. The corresponding analyses are presented in Figures 5, 6 and S3–S6.

Table S1. Values of the contour length *L*, the translational diffusion coefficient D_T, the rotational correlation time τ_R, the rotational diffusion coefficient D_R, and the calculated length *l* as determined by an analysis of the wide-field fluorescence microscopy movies. To calculate *l*, we assumed a rod shape and neglected end-correction coefficients for the microgel chains a, b and c (see also Figure 3 of the paper).

Determined chain parameters	Chain a movie 1	Chain a movie 2	Chain a movie 3
Contour length *L*/μm	3.8	11.4	7.6
D_T/m²·s⁻¹	1.53×10^{-13}	0.81×10^{-13}	1.05×10^{-13}
τ_R/s	9.8	19.4	42.9
D_R/s⁻¹	0.10	0.051	0.023

5. Additional Examples of Image Series from Confocal Microscopy

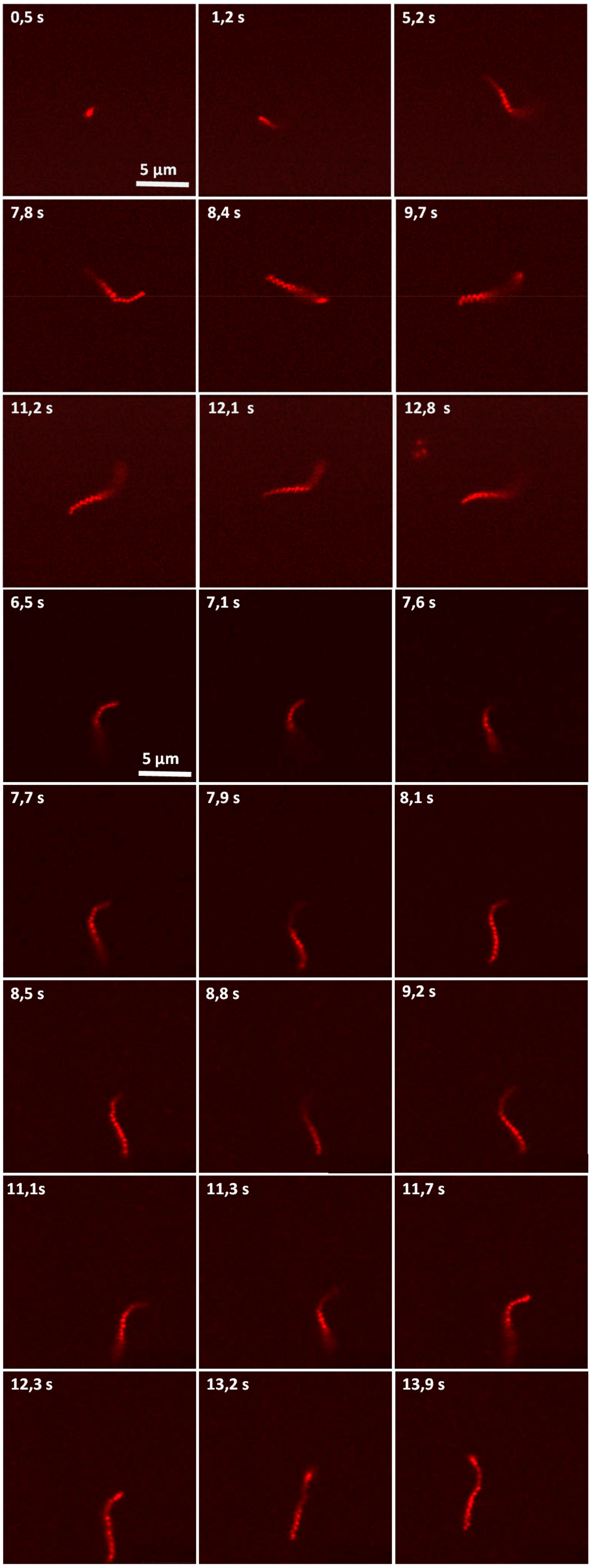

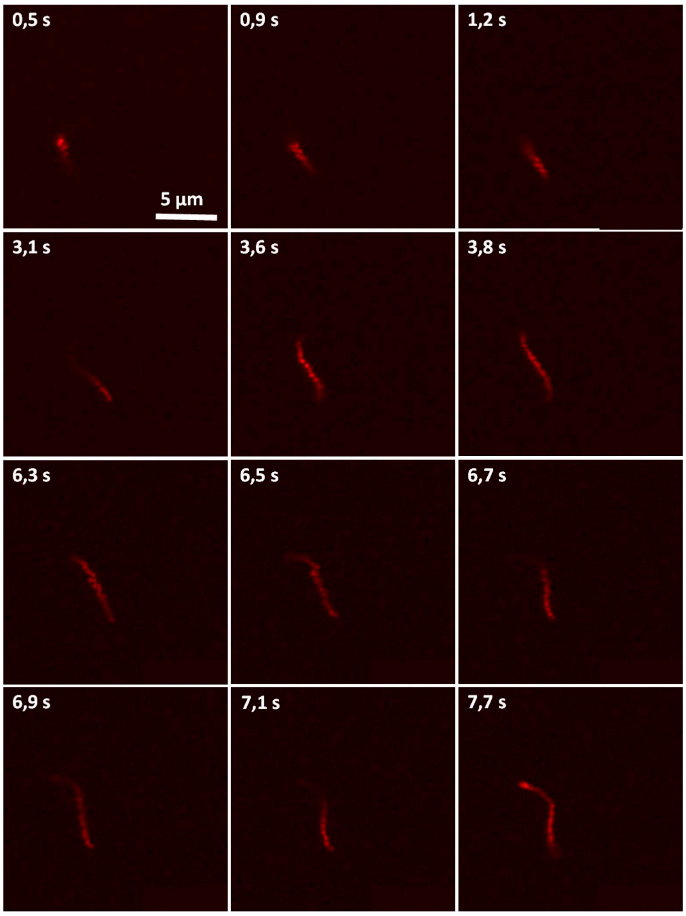

Figure S7. Three examples of freely moving chains (with adsorbed rhodamine B) observed by confocal fluorescence microscopy.
